# Evaluation of performance of leading algorithms for variant pathogenicity predictions and designing a combinatory predictor method: application to Rett syndrome variants

**DOI:** 10.7717/peerj.8106

**Published:** 2019-11-27

**Authors:** Satishkumar Ranganathan Ganakammal, Emil Alexov

**Affiliations:** 1 Department of Healthcare Genetics, School of Nursing, Clemson University, Clemson, SC, USA; 2 Department of Physics, Clemson University, Clemson, SC, USA

**Keywords:** Pathogenic mutations classification, Disease-causing mutations, Machine learning, Rett syndrome, DNA variants, Variant pathogenicity predictors

## Abstract

**Background:**

Genomics diagnostic tests are done for a wide spectrum of complex genetics conditions such as autism and cancer. The growth of technology has not only aided in successfully decoding the genetic variants that causes or trigger these disorders. However, interpretation of these variants is not a trivial task even at a level of distinguish pathogenic vs benign variants.

**Methods:**

We used the clinically significant variants from ClinVar database to evaluate the performance of 14 most popular in-silico predictors using supervised learning methods. We implemented a feature selection and random forest classification algorithm to identify the best combination of predictors to evaluate the pathogenicity of a variant. Finally, we have also utilized this combination of predictors to reclassify the variants of unknown significance in MeCP2 gene that are associated with the Rett syndrome.

**Results:**

The results from analysis shows an optimized selection of prediction algorithm and developed a combinatory predictor method. Our combinatory approach of using both best performing independent and ensemble predictors reduces any algorithm biases in variant characterization. The reclassification of variants (such as VUS) in MECP2 gene associated with RETT syndrome suggest that the combinatory in-silico predictor approach had a higher success rate in categorizing their pathogenicity.

## Introduction

With advances in genomic sequencing, molecular genomics has quickly become a standard in clinical genetics and diagnostics. Molecular genetic testing involves the identification of variants in clinical actionable regions of a single gene or multiple genes that can cause a genetic disorder. Gene based molecular diagnostics tests have the ability to contribute to a wide range of testing types such as screening for specific conditions such as autism or cancer. As the degree of severity of the disease depends on the genes affected and its associated variants along with their phenotype characteristics, multi-gene screening provides a gateway to analyze a set of genes that are found to be associated with a specific or multiple phenotype all at once (Niroula & Vihinen, 2017). The field of genomics has provided a continuous evolving platform to decode the human genome.

Advances in molecular genomic technologies such as next-generation sequencing that includes whole exome sequencing (WES) and whole genome sequencing (WGS) have aided in screening multiple genes in an instance to identify several single nucleotide variants (SNVs) that contribute to a disease (Genetic Alliance; District of Columbia Department of Health, 2010). Many disease phenotypes have been linked to the missense variants. They are considered as most clinically relevant as they alter the amino acid encoding a protein that can affect the gene function. These SNVs are not only involved in disease causing but also play an important role in altering biological processes such as transcriptional regulation, splicing (Thusberg & Vihinen, 2009).

The SNVs identified from methods like WGS or WES are evaluated based on the metadata obtained from variant annotation process that is a part of the bioinformatics workflow using information from various data sources such as Online Mendelian Inheritance in Man (Hamosh et al., 2005) and Human Gene Mutation Database (Stenson et al., 2014) databases. Using this annotated information, the variants are classified into pathogenic, benign, likely pathogenic or benign, variant of uncertain significance (VUS) and incidental findings based on American College of Medical Genetics and Genomics (ACMG) recommendations (Richards et al., 2015). The process of characterizing a particular variant’s clinical relevance such as pathogenic (disease causing) or nonpathogenic (non-disease causing or benign) poses a challenge due to issues such as differences in information from the bioinformatics workflows, limited availability of computational resources and the lack of trained professionals, despite that various computational algorithms have been developed to predict the clinical pathogenicity of variant based features such as homology, conservation based on evolution, protein function etc. (Dong et al., 2015).

In this study we employ supervised learning strategies on variants from ClinVar database (Landrum et al., 2016) to evaluate and identify the best combination of in-silico prediction algorithms to implement a best performing combinatory predictor method to characterize its pathogenicity. The results can provide a framework for bioinformaticists and molecular genomicists to review the clinical relevance of a variant by minimizing both false positive and false negative predictions. This also provides a benchmark set of predictors that could be used to determine and reclassify the variants of unknown clinical significance.

### Overview of in-silico prediction algorithms

Most of the computational methods use prediction features and then identify and implement the best performing algorithm on a training data set to classify and curate the pathogenicity of variant (Vihinen, 2012). Some predictors aim at predicting if the variant is disease-causing, others focus on predicting molecular effects caused by the mutation (Kucukkal et al., 2015; Petukh, Kucukkal & Alexov, 2015; Peng & Alexov, 2016; Peng, Alexov & Basu, 2019). ACMG has drafted policy statement and guidelines for categorizing variants according to which a variant should have multiple computational evidence to support its deleterious effect from these computational algorithms or predictors. There are two types of in-silico predictors (Table 1), (i) Independent predictors (SIFT (Sorting Intolerant From Tolerant) (Ng & Henikoff, 2003), Polyphene2 (Polymorphism Phenotyping V-2) (Adzhubei et al., 2010), PROVEAN (Protein Variation Effect Analyzer) (Choi et al., 2012), Likelihood ratio test (LTR) (Chun & Fay, 2009), Mutation Taster (Schwarz et al., 2010), Mutation Assessor (Reva, Antipin & Sander, 2011), FATHMM (Shihab et al., 2014), VEST3 (Carter et al., 2013)) employs computational algorithm that considers unique features to determine the deleteriousness of a variant (ii) Ensemble predictor (REVEL (Ioannidis et al., 2016), Mendelian Clinically Applicable Pathogenicity (M-CAP) (Jagadeesh et al., 2016), MetaLR (Dong et al., 2015), MetaSVM (Dong et al., 2015), Combined Annotation Dependent Depletion (CADD) (Rentzsch et al., 2019), Eigen (Ionita-Laza et al., 2016)) includes computation algorithms that uses collected features from various independent predictors to determine the pathogenicity of a variant. These prediction methods use different computational algorithms (Markov model, evolutionary conservation, random forest etc.) so in order to eliminate the algorithm biases it is advisable to use multiple prediction algorithms for variant evaluation (Richards et al., 2015).

**Table 1:** Description of In-silico predictors evaluated. Brief description of the fourteen in-silco predictors (both independent and empirical predictors) used in this study with pathogenicity cutoffs values.

Predictor	Description	Pathogenicity cutoff
SIFT	It uses MSA methodology that determines the probability that a missense variant is tolerated conditional on the most frequent amino acid being tolerated (Ng & Henikoff, 2003)	<0.049
Polyphen2	It calculates the normalized accessible surface area and changes in accessible surface propensity resulting from the amino acid substitution (Adzhubei et al., 2010)	>0.447
LTR	It uses heuristic methods to identify mutations that disrupt highly conserved amino acids within protein-coding sequences (Chun & Fay, 2009)	NA
Mutation taster	It uses naive Bayes classifier to evaluate the pathogenicity of a variant based on information available from various databases (Schwarz et al., 2010)	>0.5
Mutation assessor	It uses the concept of evolutionary conservation that affects amino acid in protein homologs (Reva, Antipin & Sander, 2011)	>1.935
FATHMM	It uses Hidden Markov Models (HMM) to assess the functionality of the candidate variant by incorporating a disease-specific weighting scheme (Shihab et al., 2014),	<−1.151
PROVEAN	It uses the concept of pairwise sequence alignment scores to predict the biological effect on the protein function (Choi et al., 2012)	<−2.49
VEST3	It uses supervised learning method utilizing *p*-values generated by gene prioritization method to assess the functionality of mutations (Carter et al., 2013)	NA
**Empirical or meta in-silico predictors**		
MetaSVM	It uses support vector machine approach on the previous generated scores (Dong et al., 2015)	>0
MetaLR	It uses logistic regression model on the previous generated scores (Dong et al., 2015)	>0.5
M-CAP	It uses gradient boosting trees method to analyze interactions between features to determine variant pathogenicity (Jagadeesh et al., 2016)	NA
REVEL	It combines all results from available prediction tools by using them as features to access the pathogenicity of a variant (Ioannidis et al., 2016)	>0.75
CADD	It uses a c-score obtained by the integration of multiple variant annotation resources (Rentzsch et al., 2019)	>19
Eigen	It uses a supervised approach to derive the aggregate functional score from various annotation resources (Ionita-Laza et al., 2016)	NA

## Materials and Methods

### Dataset

The ClinVar variant data is downloaded in a tab separated format from the ClinVar database (a public archive with interpretations of clinically relevant variants) FTP site available at https://www.ncbi.nlm.nih.gov/clinvar/ (Landrum et al., 2016). A customized perl script was written to parse SNVs corresponding to the “GRCh37” build. Table 2 shows the counts variants from the cleaned data categorized with clinical relevance such as “pathogenic,” “benign,” “likely pathogenic,” “likely benign” and VUS. For this paper, we choose only “pathogenic” and benign” and then apply purging procedure to eliminate cases where there is no strong evidence to be classified as “pathogenic” or “benign.” The purging was done by reading number of submitters (NOS) and submitter categories (SC) and then applying the following filters: (a) for pathogenic mutations taken from ClinVar we require that NOS > 2 & SC = 3; (b) for nonpathogenic/benign mutation the filter was NOS > 3 & SC >= 2 (Table 3). This reduced the number of pathogenic mutations from 36,536 to 2,123, and benign mutations from 7,249 to 2,231. This reduced dataset is termed “golden set.” The purging had two outcomes: reduced the noise of potentially wrong classifications and the number of pathogenic and benign mutations became very similar. This dataset serves for two purposes (i) as cross validation data set to test the performance of all the 14 in-silico algorithms evaluated (ii) and as a training data set to aid in re-classification of the uncategorized variants types such as VUS or conflicting interpretation variants.

**Table 2:** Clinical relevance distribution of variants from ClinVar database. Counts of Single Nucleotide Variants (SNVs) from ClinVar Database (for build GRCh37) categorized based on major clinical relevance.

Clinical relevance	Total number of variants
Pathogenic	36,536
Benign	7,249
Likely pathogenic	2,105
Likely benign	17,295
Variant of unknown significance (VUS)	135,534

**Table 3:** Proposed golden dataset set. The golden data set that includes pathogenic and benign variants obtained by filtering the ClinVar SNVs (build GRCh37) based on the number of submitters (NOS) and submitter categories (SC).

Clinical relevance	Total number of variants	Criteria
Pathogenic	2,123	NOS > 2 & SC = 3
Benign	2,231	NOS > 3 & SC >= 2
Total	4,354	

Another dataset, called Rett syndrome dataset, consists of variants in MeCP2 gene that are associated with Rett syndrome (Zoghbi, 2016; Gold et al., 2017). The data was extracted from downloaded ClinVar database. This data set has all the differently clinically categorized variants that includes 64 pathogenic and one benign variant and 115 variants classified as like benign/pathogenic, VUS and conflicting interpretations. Out of the 115 variants we were able to clean up our testing to 101 variants that has associated in-silico predictor score available. They will be subjected to our best set of in-silico predictors and reclassified as pathogenic or benign.

### Feature extraction

We annotated all the variants from our prepared dataset using the dbNSFP data source v2.9.3 (https://sites.google.com/site/jpopgen/dbNSFP) (Liu, Jian & Boerwinkle, 2011). This data source includes scores from all the in-silico predictors along with allele frequency information from various population databases. A customized perl script is used to extract the consolidated in-silico scores from both dependent and independent predictors such as SIFT, Polyphen2, LTR, Mutation Taster, Mutation Assessor, FATHMM, PROVEAN, VEST3, MetaSVM, MetaLR, M-CAP, Revel, CADD, Eigen. These scores are used as features for our features ranking and performance evaluation algorithms.

### Features ranking and performance evaluation

We used the scores from the 14 in-silico predictors as features to access their performance. We evaluated the in-silico predictors on the ability to distinguishing the variants of our dataset into pathogenic or benign based on the statistics collected from the confusion matrix. We used Weka (v3.8.2) (Hall et al., 2009) to collect statistics about accuracy, sensitivity, specificity, precision, *F*-measure and Mathews correlation coefficient (MCC) calculated using the number of true positives (TP), true negatives (TN), false positives (FP) and false negatives (FN) (Table 4).

**Table 4:** Statistical measure from our supervised learning method. Various statistics values calculated from our performance evaluation and classification analysis from Weka Software.

Statistics	Formula
Sensitivity	$\frac{\rm TP}{(\rm TP + \rm FN)}$
Specificity	$\frac{\rm TN}{(\rm TN + FP)}$
Precision	$\frac{\rm TP}{(\rm TP + FP)}$
*F*-measure	$$\frac{2 \times {\rm Precision \times \rm recall}{(Precision + recall)}$$
MCC	$\frac{{\rm{TP}} \times {\rm{TN}} - {\rm{FN}} \times {\rm{FP}}}{\sqrt {({\rm{TP}} + {\rm{FN}})({\rm{TP}} + {\rm{FP}})({\rm{TN}} + {\rm{FN}})({\rm{TN}} + {\rm{FP}})}}$

### Identification of best in-silico predictor set

For the development of the best predictor set, firstly we evaluated the two class of attributes or features (independent and ensemble predictors scores) separately using the ranker attribute evaluation method. This provides with a list of the best performing in-silico algorithms. Secondly, we identified the best combination of in-silico predictors that includes top performing independent and ensemble predictors that can best classify the variant as either benign or pathogenic. The “ranker” option nested under the classifier attribute evaluation method is used for ranking features (in-silico scores), it is a fast and precise method that considers only relevant attributes and eliminates both irrelevant and redundant features that ranking our methods based on more on correlation. Thus, the algorithm ranks the features based on their strength of classification. We also use Random forest as a classifier method along with the Ranker evaluator method to rank and evaluate the in-silico predictor based on performance.

## Results

### Selection of best classification algorithms

The predictors’ scores on the golden dataset with 4,354 variants bearing stronger evidence to be categorized as pathogenic and benign were used as an input for the machine learning algorithms. For the first step of our analysis we applied the best performing classification methods for the evaluation of our dataset. Table 5 shows the statistics. Based on our findings, we identified Random forest method as the best classifier method when compared to others such as Naïve Bayes, Classification via Regression, LibSVM with 97% accuracy.

**Table 5:** Summary of various supervised learning method. Statistics calculated on our cross-validation dataset by applying different machine learning algorithms to identify the best methods for feature evaluation.

Classification algorithm	Sensitivity	Specificity	Precision	Recall	*F*-Measure	MCC	Accuracy
Random forest	0.985	0.952	0.956	0.985	0.970	0.938	0.969
Naive Bayes	0.905	0.911	0.914	0.905	0.909	0.815	0.907
Classification via regression	0.957	0.944	0.948	0.957	0.953	0.902	0.951
LibSVM	0.940	0.930	0.934	0,940	0.937	0.870	0.953

### Evaluation of the performance of in-silico prediction methods

**Figure 1:**
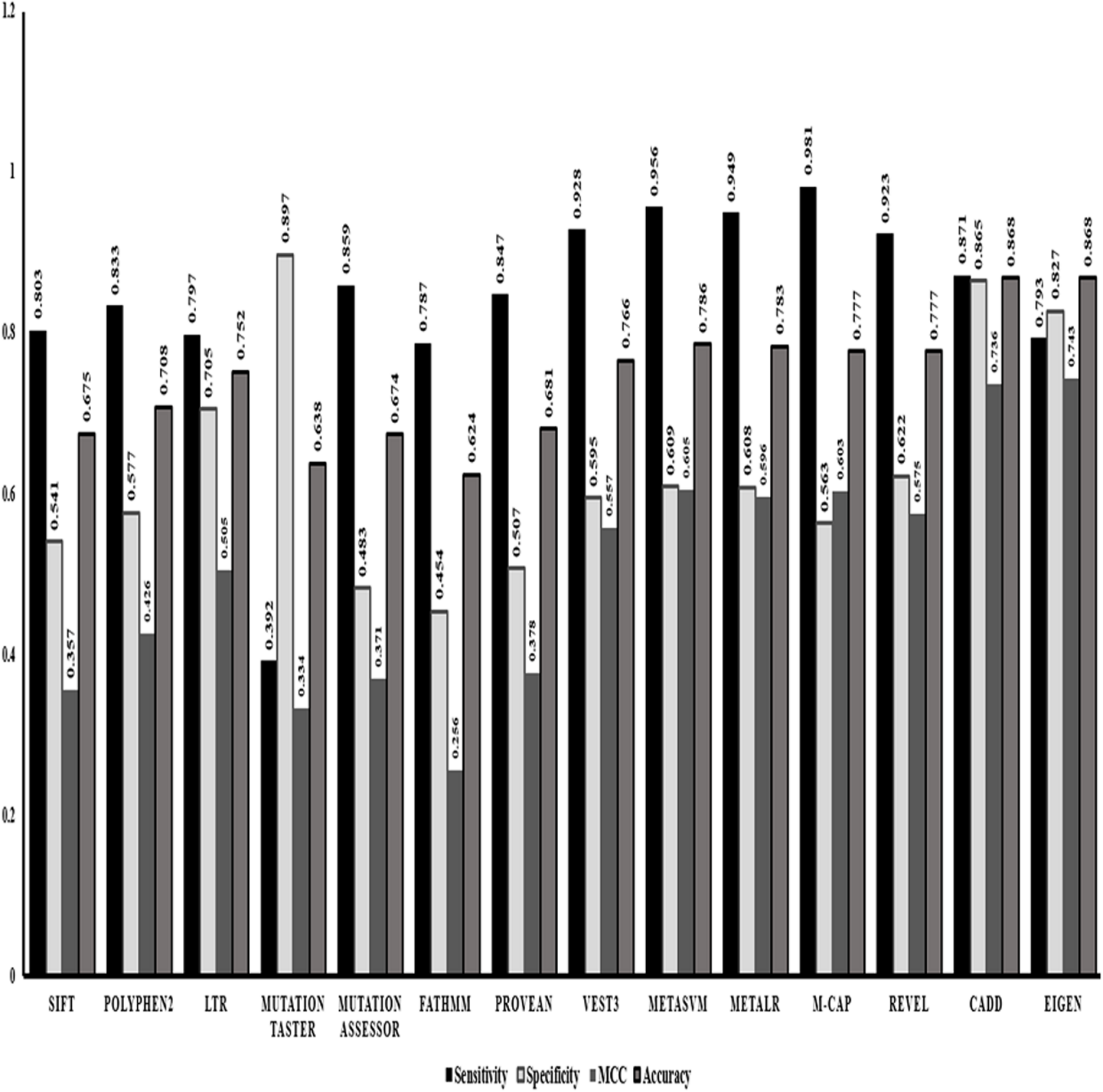
Performance evaluations of 14 in-silico predictors. The graphical representation of the major statistics obtained from the evaluation of all 14 in-silico predictors.

After identifying Random forest as the best classifier method, we evaluated the performance of the in-silico predictors separately based on the strength of classification of a variant into benign or pathogenic class. Comparing the statistics obtained from the classification method (Fig. 1) we identified that ensemble or the dependent predictor out performed almost all the independent predictor algorithms with higher sensitivity and accuracy. VEST3 is the only independent predictor that has seems to have a sensitivity and accuracy comparable to the ensemble predictors. Even the comparison based on MCC values that is used for evaluation of imbalanced data displays the same trend where the ensemble predictors fared better than the independent predictors.

**Figure 2:**
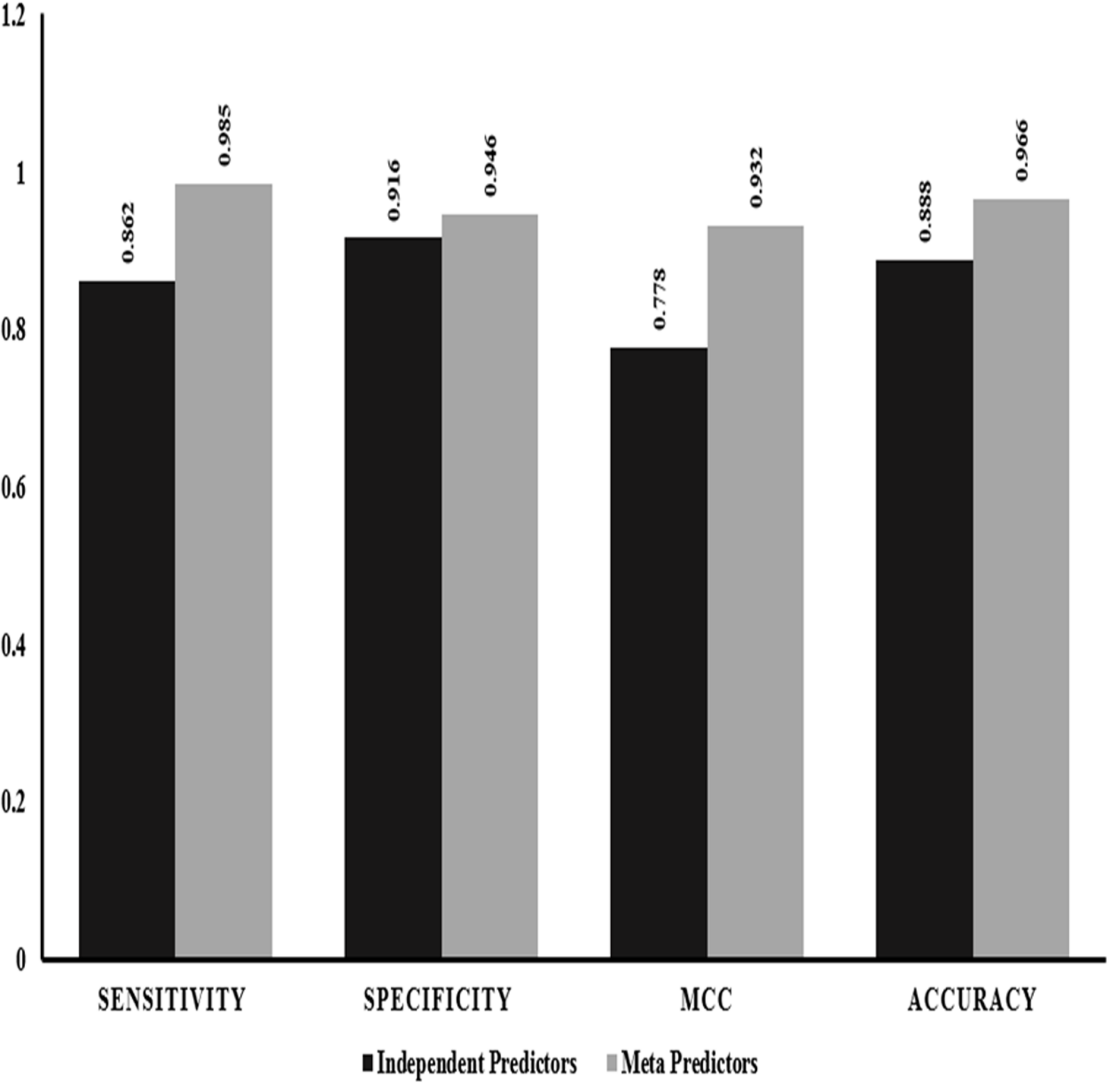
Performance comparison: independent vs empirical in-silico predictors. The graphical representation of the major statistics obtained from the evaluation of both independent (solid bars) and ensemble (grey bars) predictors.

We also performed evaluation of dependent predictors and independent/ensemble predictors separately using the 10-fold cross validation strategy by implementing random forest method to check for the best performing classification predictors based on accuracy and other statistics of predictions. The ensemble predictors outperform the independent predictors with an accuracy of ~97% along with higher sensitivity, specificity and MCC values (Fig. 2).

### Identification of the best performing in-silico predictor set

The results from the feature evaluation of the ranking attribute methods for both the independent and ensemble predictors separately identified the best in-silico predictors that can now be used to classify the variant data better into pathogenic and benign sub-categories.

VEST3, LTR, Polyphene2 and PROVEAN are identified as the top four ranked independent in-silico predictors and CADD, Eigen, MetaSVM and REVEL are identified as the top four ranked ensemble in-silico predictors. Figure 1 shows that these in-silico predictors did show higher sensitivity, MCC and accuracy trends too compared to the other in-silico predictors in their respective category.

**Figure 3:**
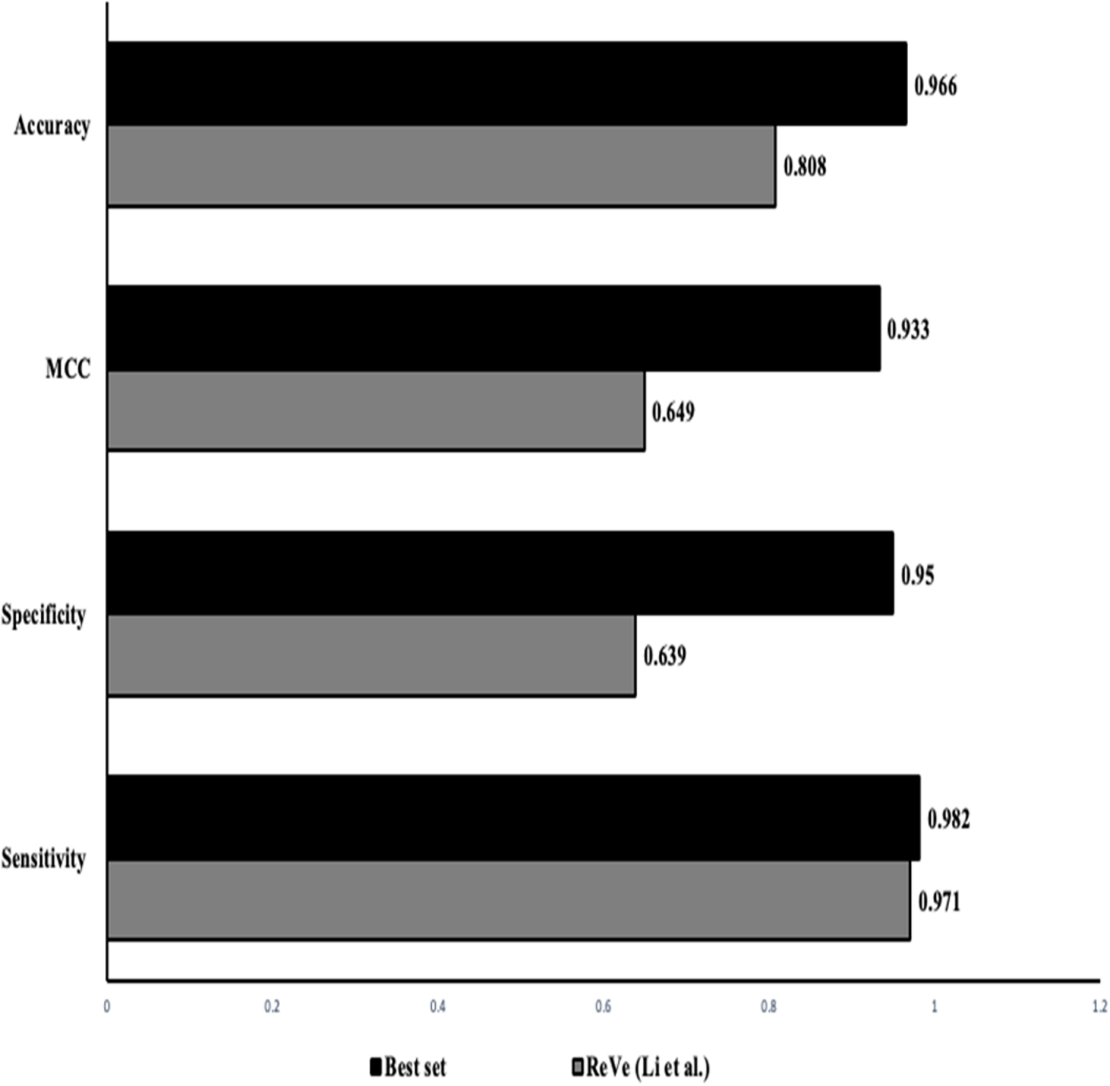
Performance comparison: our approach vs ReVe. Comparison of the statistics obtained from the proposed combined set of independent (VEST3, LTR, Polyphen2 and PROVEAN) and ensemble or dependent (CADD, Eigen, MetaSVM and REVEL) predictors (solid bars) to the combination of REVEL and VEST as proposed by Li et al. (2018) (grey bars).

The classification of SNVs with the top four predictors from both independent and ensemble predictor categories together shows a better and stronger evidence to evaluate variant pathogenicity. The higher rate of observed accuracy, sensitivity and the MCC statistics from the classification of our data with just the selected eight features proves that our combined in-silico predictor set can be highly reliable with comparatively minimal algorithmic biases (Table 6). Figure 3 shows that our identified combination of the predictors outperforms previously proposed combination proposed by Li et al. (2018) that includes just two predictors (REVEL & VEST3).

**Table 6:** Summary statistics of our combinatory approach. Statistics obtained by applying our classifier to the golden dataset with proposed combined set of independent (VEST3, LTR, Polyphen2 and PROVEAN) and ensemble or dependent (CADD, Eigen, MetaSVM and REVEL) predictors.

Predictors	Classification algorithm	Sensitivity	Specificity	Precision	Recall	*F*-Measure	MCC	Accuracy
VEST3, LTR, Polyphene2, PROVEAN. CADD, Eigen, MetaSVM and REVEL	Random forest	0.982	0.950	0.954	0.982	0.968	0.933	0.966

### Rett syndrome variants

The 197 variants associated with Rett syndrome collected from the ClinVar database includes 101 variants which are categorized into likely benign/pathogenic, uncertain significant (VUS) and conflicting interpretations of pathogenicity classes, along with 64 pathogenic and 1 benign variant.

We used our best in-silico predictor set to reclassify the above (64 pathogenic and 1 benign) variants either as pathogenic or benign with an average classification accuracy of 89% and 100%, respectively (Table 7). This assures the performance of the proposed set of in-silico predictors. Furthermore, Table 7 summarizes the fringe variants re-classified, with our best set of in-silico predictors out of which 60% was classified into pathogenic category and 40% was classified into benign category.

**Table 7:** Reclassification of the MECP2 variants. Variants that was previously classified as likely benign/pathogenic, uncertain significant (VUS) and conflicting interpretations of pathogenicity classes was reclassified using our golden dataset (as training dataset) along with benchmarking against “pathogenic” and “benign” mutations.

Clinical significance	Total variants	Classification on best in-silico predictors		Success rate
		Benign	Pathogenic	
Pathogenic	64	7	57	89%
Benign	1	1	0	100%
Likely Benign	10	9	1	NA
Likely pathogenic	11	2	9	NA
Uncertain significance	69	25	44	NA
Conflicting interpretation	11	5	6	NA

## Discussion

The advances in computer algorithm had been widely utilized in the evaluation of the pathogenicity of a variant. We evaluated the performance of 14 prominent in-silico computational algorithm methods with 4354 SNVs from purged ClinVar database (golden dataset). We also evaluated the performance of eight independent and six ensemble predictors that led us to identify the best combination of in-silico predictors that can categorize variants into either disease causing or not. Our initial investigation revealed that the ensemble prediction algorithms outperformed the independent algorithm with a higher accuracy of variant categorization.

Our individual assessment of the in-silico prediction methods shows that VEST3 is the best performing independent predictor method with high accuracy of classifying variants. However, the main limitation is that this algorithm is based on prioritization of missense variants thus creating a partizanship biases in evaluating the non-missense variants. Similarly, Eigen and CADD are the best performing empirical algorithms based on accuracy of classification which is also highly influenced by the algorithm constrain that decreases the sensitivity and specificity of variant characterization. The pathogenicity of variant can also be associated with the different scoring strategies used by either supervised or unsupervised learning methods. This provides us with a strong platform to investigate a combinatory approach that includes both dependent and empirical predictors to evaluate the pathogenicity of a variant.

Although there are many studies that performed comparative investigation to identify the best performing in-silico predictor method, (Li et al., 2018) in their study displayed the effectiveness of a combinatory approach where the combination of two in-silico predictors, VEST3 and REVEL displayed better overall performance in characterization of clinically significant missense variants. This combination when extended to both missense and non-missense variants displayed less accuracy, sensitivity and specificity compared to the just the empirical predictors. The results from feature selection analysis identified the best combination of independent and empirical predictors that can distinguish and characterize the variant pathogenicity.

To select the best combination of predictors, we first used the attribute ranking method for overall ranking of the predictors, followed by combination predictors using each in-silico predictor sub-group (independent and ensemble). We picked the combination with the least number of predictors with the highest prediction accuracy. Based on the statistics obtained (a few combinations are highlighted in Table S1) the combination of top four methods had the highest accuracy, sensitivity and MCC values. Thus we combined the top four performing in-silico predictors from both the empirical and the conventional methods (independent methods: VEST3, LTR, Polyphene2 PROVEAN; ensemble methods: CADD, Eigen, MetaSVM and REVEL) that yielded an accuracy of 97% which is similar to the accuracy yielded by just the empirical predictors, while providing information on a variant with a minimized biased evaluation. It even outperformed the ReVe (Revel and Vest3) combination from Li et al. (2018) with higher sensitivity and accuracy measures (Fig. 3) (Table S1). During our selection we noticed that the empirical predictors MetaLR and REVEL both exhibited similar accuracy, sensitivity and specificity but we included REVEL into our set as it has been validated with larger sample set and has exhibited greater performance in classifying missense variants.

After we demonstrated that the algorithm performs well, it was applied to reclassify the variants associated with RETT syndrome listed as uncertain or conflicting clinical significance. This reclassified set can be used to guide further clinical investigation for mutants linked with Rett syndrome along with studies about the effects of mutation on wild type characteristics of the corresponding protein.

## Conclusions

In summary, our combinatory approach of using both best performing independent and empirical predictors reduces any algorithm biases in variant characterization. Our robust training dataset composed of ClinVar variants filtered based on strong evidences for pathogenic and benign characteristics can reduce the false positive and true negative results. Also, similar filtering approaches for data preparation can be used in development of new methods for accessing the functional effect of a variant. Though in-silico predictors are just one of data point in evaluation of variant pathogenicity along with other information such as allele frequency, our predictor set will aid in consolidated balanced prediction thus increases the confidence of evaluation. This also could provide sufficient evidence for clinical genomicist and researchers to understand and evaluate the pathogenicity of variants whose clinical relevance is unknown.

## Supplemental Information

10.7717/peerj.8106/supp-1 Supplemental Information 1 Re-classified Rett syndrome variants.

10.7717/peerj.8106/supp-2 Supplemental Information 2 Training dataset.

10.7717/peerj.8106/supp-3 Supplemental Information 3 Perl script to parse ClinVar entries.

10.7717/peerj.8106/supp-4 Supplemental Information 4 Golden dataset.

## Additional Information and Declarations

### Data Availability

The following information was supplied regarding data availability: We used Clinvar data: https://www.ncbi.nlm.nih.gov/clinvar/. The data we generated is available at http://compbio.clemson.edu.
